# Carrizo citrange Plants Do Not Require the Presence of Roots to Modulate the Response to Osmotic Stress

**DOI:** 10.1100/2012/795396

**Published:** 2012-07-31

**Authors:** Rosa M. Pérez-Clemente, Almudena Montoliu, Sara I. Zandalinas, Carlos de Ollas, Aurelio Gómez-Cadenas

**Affiliations:** Department of Agricultural Sciences, Universitat Jaume I, Campus Riu Sec, 12071 Castelló de la Plana, Spain

## Abstract

The study of the effects of a specific stress condition on the performance of plants grown under field conditions is difficult due to interactions among multiple abiotic and biotic factors affecting the system. *In vitro* tissue-culture-based techniques allow the study of each adverse condition independently and also make possible to investigate the performance of genotypes of interest under stress conditions avoiding the effect of the root. In this paper, the response of Carrizo citrange, a commercial citrus rootstock, to osmotic stress was evaluated by culturing *in vitro* intact plants and micropropagated shoots. The osmotic stress was generated by adding two different concentrations of polyethyleneglycol to the culture media. Different parameters such as plant performance, organ length, antioxidant activities, and endogenous contents of proline, malondialdehyde, and hormones were determined. Differently to that observed under high salinity, when subjected to osmotic stress conditions, Carrizo citrange showed increased endogenous levels of MDA, proline, and ABA. These results evidence that the mechanisms of response of Carrizo citrange to saline or osmotic stress are different. The presence of roots was not necessary to activate any of the plant responses which indicates that the organs involved in the stress perception and signaling depends on the type of adverse condition to which plants are subjected.

## 1. Introduction

Water is essential for plant growth and a necessary component of most physiological processes. Drought and other abiotic factors often decrease soil water potential and difficult plant water uptake. Water deficiency in a plant, which results from an imbalance between water uptake and loss, leads to the disturbance of various physiological processes [1].

Citrus is one of the most important horticultural crops globally and is considered as being salt sensitive [2, 3]. In the Mediterranean area, low rainfall and high temperatures in summer along with the high concentrations of salts found in the irrigation water often result in agricultural crops suffering simultaneous water and salt stress [4]. Water stress in citrus reduces growth and metabolism, leading to a reduction in fruit yield and quality [5, 6].

The study of the effects of a specific stress condition on the performance of plants grown under field conditions is difficult due to different uncontrolled situations such as interactions among multiple abiotic and biotic stress factors. Moreover, the measurement of root traits in field-grown plants is laborious, time-consuming, and not always possible [7].

Development of tools to separately investigate specific components of the multiple factors affecting plant development can be useful for both academic and applied research. The *in vitro* tissue culture techniques can overcome some of the above described limitations and allow growing clonal plants under controlled climatic and nutritional conditions to carry out experiments in identical conditions all year round [8]. In a previous work, we used this approach to study the performance of different citrus genotypes cultured under salt stress conditions, avoiding the effect of the root by culturing shoots of those genotypes without the root system. The method proved to be a good tool for studying biochemical processes involved in the response of citrus to salt stress [9].

Addition of sorbitol or polyethylene glycol (PEG) decreases medium water potential, inducing water stress that adversely affects both shoot and root growth of the plantlets [10]. Polymers of PEG have been used for many years, mainly because PEG molecules with a molecular weight of 6000 g/mol (PEG-6000) cannot penetrate the cell wall pores [11]. Because PEG does not enter the apoplast, water is with drawn not only from the cell but also from the cell wall. Therefore, PEG solutions mimic dry soil more closely than solutions of compounds with low molecular weights, which infiltrate the cell wall with solute [12].

Plants grown under osmotic stress conditions are seriously affected by oxidative stress. One of the earliest responses aiming water loss avoidance involves stomatal closure, which subsequently downregulates the photosynthetic machinery due to a decrease in CO_2_ uptake [13]. As a consequence, the photosynthetic electron transport chain becomes overreduced, resulting in the generation of reactive oxygen species (ROS) [14]. In plant cells, the excessive production of ROS is potentially harmful to lipids, proteins, and nucleic acids [15], whose oxidation may, in turn, lead to detrimental effects such as enzyme inhibition, chlorophyll degradation, disruption of membranes, among others [16]. Malondialdehyde (MDA), a decomposition product of polyunsaturated fatty acids in biomembranes, is often used as indicator of peroxidation, and this compound accumulates under several abiotic stress conditions [17, 18]. Several enzymatic systems and antioxidant molecules are responsible to counteract the deleterious effects of ROS. The first enzymatic step in the detoxifying process is the superoxide dismutase activity (SOD), which catalyses the conversion of O_2_^•−^ to H_2_O_2_. The H_2_O_2_ is then reduced to water by the enzymatic activities ascorbate peroxidase (APX), which utilizes ascorbate as the specific electron donor, and catalase (CAT), which does not require any reducing equivalent [16].

Besides the activation of different detoxification systems, plant tissues accumulate compatible solutes [19] such as the amino acid proline which is one of the most widespread osmoprotectants [20] to neutralize osmotic stress; in addition to its role for osmotic adjustment under stress conditions, its capacity for quenching reactive oxygen radicals may help cells to overcome oxidative damage caused by water deficit [21]. In this respect, proline has been proposed to stabilize DNA, membranes, and proteins [22]. Moreover, the role of proline in plant responses to oxidative stress has been extensively shown by experiments utilizing exogenous application of this amino acid [23] or by genetic manipulation of its metabolism; it has been reported that transgenic “Swingle” citrumelo plants overexpressing Δ1-pyrroline-5-carboxylate synthetase gene (P5CS), involved in the first two steps of proline biosynthesis, exhibited better osmotic adjustment, and tolerated longer period in severe conditions of drought stress than wild-type plants [18].

The present study was designed to evaluate the response of Carrizo citrange, a citrus rootstock widely used in citriculture worldwide, to moderate and severe osmotic stress conditions. To depict the specific effect of this abiotic stress on plant physiology without the interferences of other factors, an *in vitro* tissue culture system was used. The osmotic stress was generated by using two different concentrations of PEG. Stress impact on the plant was evaluated by measuring growth and levels of two metabolites, proline and MDA, as stress markers. Physiological responses to osmotic stress were evaluated by measuring plant hormonal concentration and different antioxidant activities.

## 2. Materials and Methods

### 2.1. Plant Materials and Treatments

Carrizo citrange (*C. sinensis *L. Osb. x *P. trifoliata *L. Raf.) seeds were peeled, and after removing their both seed coats, they were disinfected for 10 min in a 0.5% (vol/vol) sodium hypochlorite solution containing 0.1% (vol/vol) Tween-20 wetting agent and rinsed three times with sterile distilled water. Seeds were sown individually in 25 × 150 mm culture tubes with 25 mL of germination medium (GM) consisting of Murashige and Tucker (MT) salt solution [24], 100 mg/L *i*-inositol, 1 mg/L pyridoxine-HCl, 0.2 mg/L thiamine-HCl, 1 mg/L nicotinic acid, and 30 g/L sucrose. The pH was set at 5.7 ± 0.1 with 0.1 N NaOH before autoclaving. The medium was solidified by the addition of agar (Pronadisa, Madrid, Spain). The cultures were maintained at 26°C, first in darkness for 2 weeks and then with a 16 h photoperiod and illumination of 45 mmol m^−2^ s^−1^. Once root system reached 3 cm length, plants were used as plant material to carry out the osmotic stress experiments.

Polyethylene glycol (PEG-6000, Panreac, Barcelona, Spain) was added to GM at two concentrations to achieve medium osmotic potential of −0.75 and −1.5 MPa, according to Michel and Kaufmann [25]. All the experiments were performed using liquid GM medium supplemented with PEG when necessary: control medium (GM), moderate-stress condition (osmotic potential of −0.75 MPa, PEG075), and severe-stress condition (osmotic potential of −1,50 MPa, PEG150). Plants were maintained in the culture conditions described above throughout experimental period. 

Another set of experiments was performed using Carrizo citrange micropropagated shoots. Both, establishment of cultures from greenhouse growing plants and the micropropagation, they were performed following the protocol described in Montoliu et al. [9]. When shoots reached 15 mm length, they were excised and used as plant material in successive experiments.

The culture medium (MT), consisted of inorganic salts of Murashige and Skoog [26], 100 mg/L *i*-inositol, 1 mg/L pyridoxine-HCl, 0.2 mg/L thiamine-HCl, 1 mg/L nicotinic acid, and 30 g/L sucrose supplemented with 0.2 mg/L of gibberellic acid and 0.2 mg/L 6-benzylaminopurine. The pH was set at 5.7 ± 0.1 with 0.1 N NaOH before autoclaving. 

As described above, for the osmotic stress treatments, different concentrations of PEG-6000 were added to MT medium to achieve osmotic potentials of –0.50 (PEG050, moderate-stress condition) and –0.75 MPa (PEG075, severe-stress condition). Shoots grown on MT were used as controls.

Samples of shoots and roots were taken for analyses at 10, 20, and 30 days after the plants were transferred to treatment media. Shoots and roots were collected, rinsed with distilled water to eliminate any residue, and frozen in liquid nitrogen. Plant material was kept at −80°C until further analyses.

### 2.2. Visible Symptoms of Plant Damage

The presence of yellowish spots at the leaf tip that progressively led to severe burning injuries was considered to be a good visible estimate of osmotic stress-induced damage to leaves. The number of damaged leaves was regularly recorded during the experimental period and expressed as a percentage of the total number of leaves. Plants showing a percentage of damaged leaves equal to or over 50% and or damage in the root (considered this as necrosis at the root apex) were considered ‘‘affected” for the osmotic treatment. Shoot and root length were also recorded after 10, 20, and 30 days of treatment.

### 2.3. Malondialdehyde Concentration

Malondialdehyde concentration was measured following the procedure described in Hodges et al. [27]. Plant material (root or shoot tissue) was homogenized in 5 mL of 80% cold ethanol (Panreac, Barcelona, Spain) using a tissue homogenizer (Ultra-Turrax; IKA-Werke, Staufen, Germany). Homogenates were centrifuged at 4°C to pellet debris and different aliquots of the supernatant were mixed either with 20% trichloroacetic acid (TCA) (Panreac) or a mixture of 20% TCA and 0.5% thiobarbituric acid (Sigma-Aldrich, Madrid, Spain). Both mixtures were allowed to react in a water bath at 90°C for 1 h. After this time, samples were cooled down in an ice bath and centrifuged. Absorbance at 440, 534, and 600 nm was read in the supernatant against a blank. The MDA concentration in the extracts was calculated as in Arbona et al. [17].

### 2.4. Antioxidant Enzyme Activity

Enzyme assays were performed as described in Arbona et al. [2]. Briefly, 0.5 g of frozen plant material was extracted in 2.5 mL of PBS using sea sand as an abrasive. The homogenate was filtered through two layers of muslin cloth. The different buffers used for enzyme extraction were for APX, 50 mM PBS pH 7.1 supplemented with 1 mM sodium ascorbate, 0.1 mM EDTA, and two drops of Triton X-100 (Panreac) and for CAT was 50 mM PBS pH 6.8. The APX activity (EC 1.11.1.11) was determined following the depletion in absorbance at 290 nm because of ascorbate consumption. CAT (EC 1.11.1.6) was assayed using the hydrogen peroxide-dependent reduction of titanium chloride. Protein content in extracts was assessed by means of the protein-dye binding method using Coomassie blue G-250 (Sigma-Aldrich). Enzyme activity was expressed as arbitrary units per mg protein.

### 2.5. Proline Content

Proline content was analyzed following the procedure described in Arbona et al. [2]. Briefly, 0.05 g of frozen plant tissue (shoot or root) was homogenized in 5% sulphosalicylic acid (Panreac) using a tissue homogenizer (Ultra-Turrax). After extraction, homogenates were centrifuged to pellet cell debris at 4°C and 1 mL aliquot of the supernatant was combined with an equal volume of glacial acetic acid (Panreac) and ninhydrin reagent. This mixture was boiled in a water bath for 1 h and then cooled in an ice bath. The solution was partitioned against 2 mL of toluene (ACS grade; Panreac) and absorbance at 520 nm measured in this organic layer. A calibration curve was performed using commercial proline as a standard (Sigma-Aldrich).

### 2.6. Hormone Analyses

Plant hormones were analyzed by HPLC coupled to tandem mass spectrometry as described in Durgbanshi et al. [28] and Arbona and Gómez-Cadenas [29]. Briefly, frozen plant material (shoot or root) was ground to a fine powder with a prechilled mortar and a pestle and then 0.5 g of powdered tissue was extracted in ultrapure water using a tissue homogenizer (Ultra-Turrax). Before extraction, samples were spiked with 100 ng of [^2^H_6_]-ABA, 100 ng of [^2^H_4_]-SA, and 100 ng of dihydrojasmonic acid to assess recovery and matrix effects. After extraction and centrifugation, the pH of the supernatant was adjusted to 3.0 and partitioned twice against di-ethyl-ether (Panreac). The organic layers were combined and evaporated in a centrifuge vacuum evaporator (Jouan, Saint-Herblain, France). The dry residue was thereafter resuspended in a water:methanol (9 : 1) solution, filtered, and injected into an HPLC system (Alliance 2695, Waters Corp., Milford, USA). Hormones were then separated in a reversed-phase Kromasil 100 C18 column (100 × 2.1 mm 5-*μ*m particle size) using methanol and ultrapure water both supplemented with glacial acetic acid to a concentration of 0.05%. The mass spectrometer, a triple quadrupole (Quattro LC, Micromass Ltd., Manchester, UK), was operated in negative ionization electrospray mode and plant hormones were detected according to their specific transitions using a multiresidue mass spectrometric method [28].

### 2.7. Statistical Analyses

Data mean comparisons and regression analyses were performed with STATGRAPHICS PLUS v.5.1 software (Statistical Graphics Corporation, Herndon, VA). One-way ANOVA and comparisons between means were made following the LSD test at *P * ≤ 0.05.

## 3. Results

To study the effect of osmotic stress in citrus, Carrizo citrange genotype, a commercial rootstock sensitive to salinity and water stress, was selected. Adjustment of PEG concentration was performed to achieve medium osmotic potentials of −0,75 MPa/−1,50 MPa and −0,50 MPa/−0,75 MPa to provide moderate- and severe-osmotic stress conditions in whole plants and micropropagated shoots, respectively. These final concentrations were fixed after comparing the effect of six different PEG concentrations in the two kind of plant material in preliminary experiments (data not shown).

### 3.1. Visible Symptoms of Plant Damage

**Figure 1:**
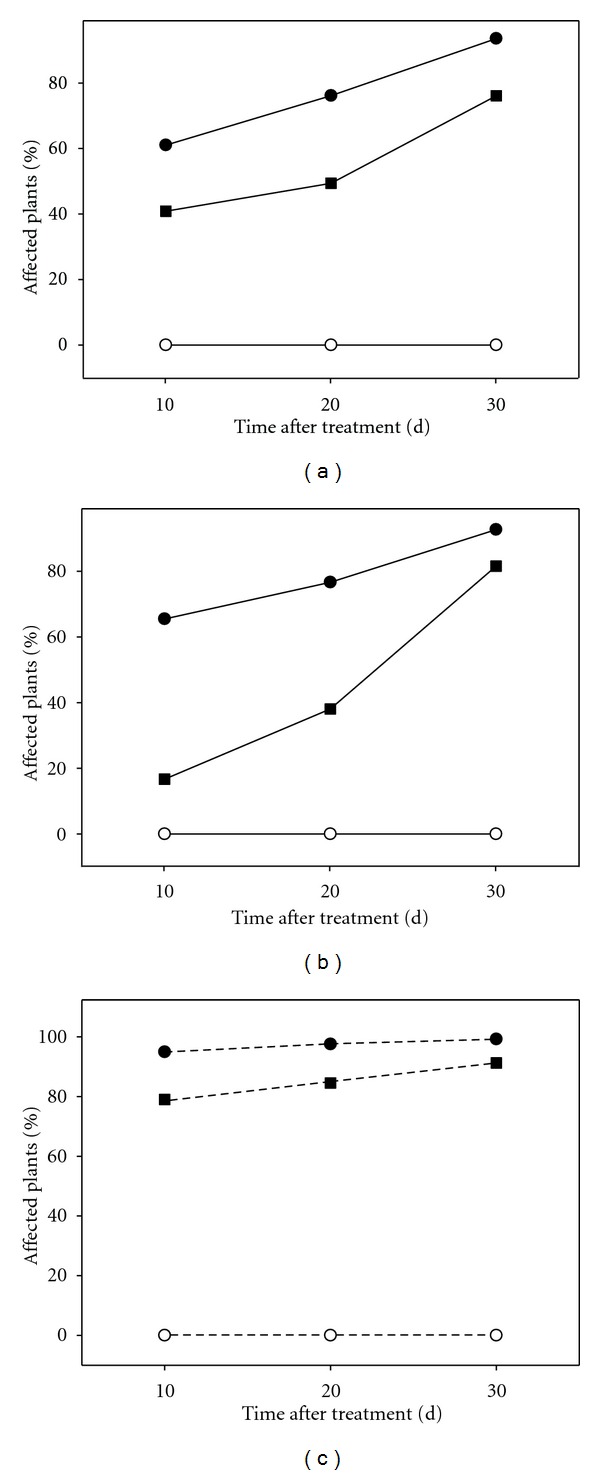
Effect of osmotic stress on plant performance in Carrizo citrange cultured *in vitro*. (a): intact plants affected in the aerial part; (b): intact plants affected in the root tissue; (c): micropropagated shoots affected. Control plants (open circle), plants cultured under moderate (filled square) or severe (filled circle) osmotic stress conditions.

From the beginning of the experiments, whole plants and micropropagated shoots cultured under osmotic stress conditions showed plant tissue damage. The percentage of affected plants and shoots was proportional to the severity of the imposed stress (Figure 1). Regardless the plant material, the percentage of affected individuals increased with time, being higher than 70% in all cases after 30 days of treatment. 

**Figure 2:**
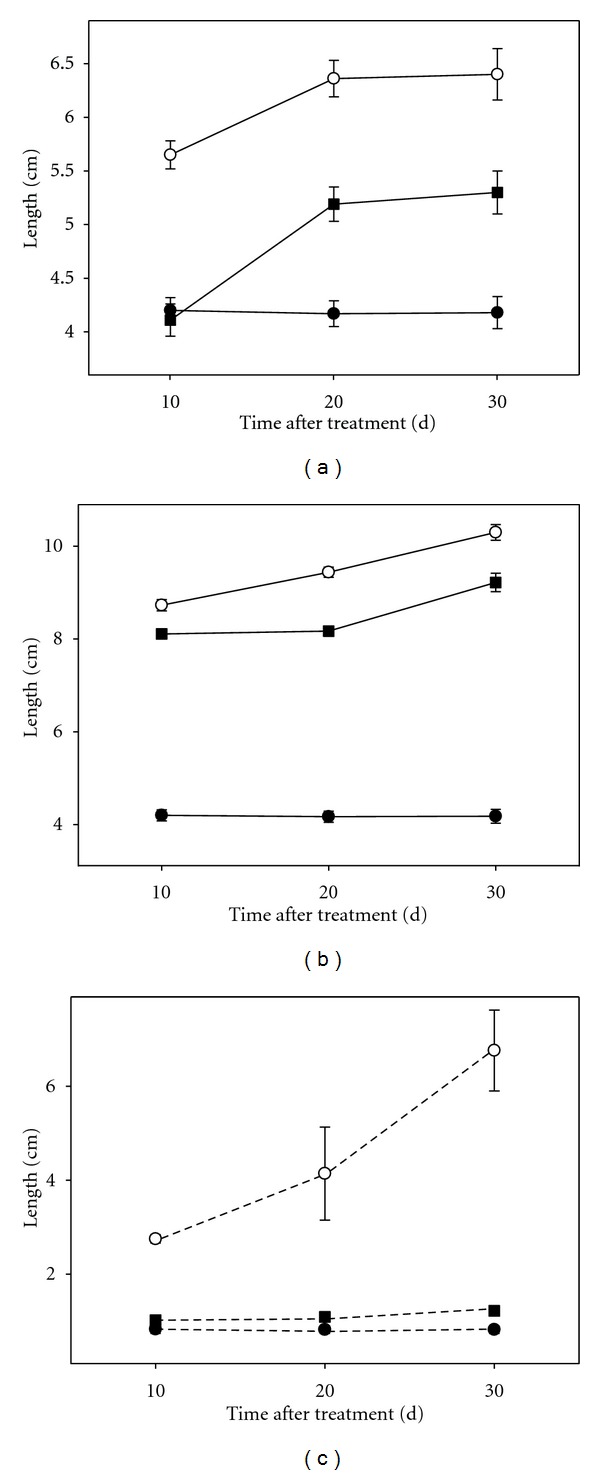
Effect of osmotic stress on organ length in Carrizo citrange cultured *in vitro*. (a): aerial part of intact plants; (b): root tissue of intact plants; (c): micropropagated shoots. Control plants (open circle), plants cultured under moderate (filled square) or severe (filled circle) osmotic stress conditions.

A significant length reduction in plants and micropropagated shoots was observed in response to the osmotic stress conditions from the first day of measurement (Figure 2). In intact plants, this reduction occurred in both organs: root and shoot (Figures 2(a) and 2(b)). Plants under severe stress did not elongate neither shoot or roots throughout the experimental period. Micropropagated shoots were also very sensitive to stress and shoots did not virtually grow under any of the two stress conditions assayed.

### 3.2. Malondialdehyde Concentration

**Figure 3:**
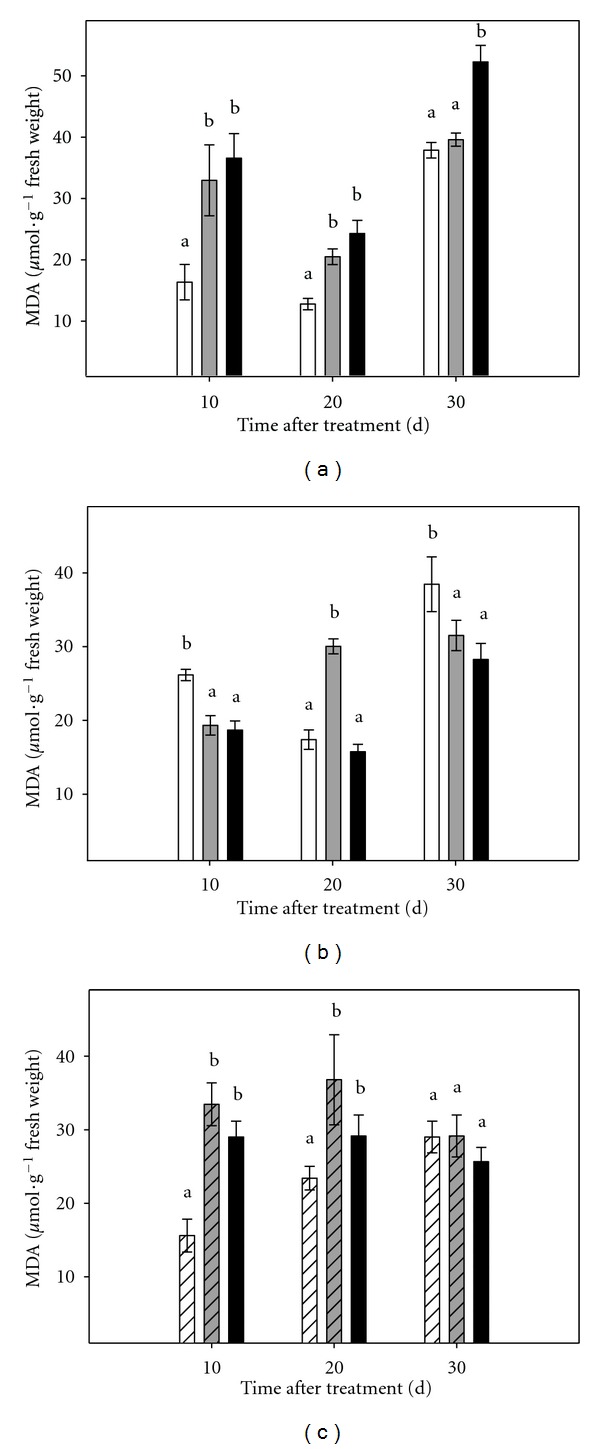
Effect of osmotic stress on MDA content in Carrizo citrange cultured *in vitro*. (a): aerial part of intact plants; (b): root tissue of intact plants; (c): micropropagated shoots. Control plants (white bar), plants cultured under moderate (grey bar) or severe (black bar) osmotic stress conditions. Each point corresponds to the average ± standard error of six independent determinations. Different letters denote statistical significance at *P *< 0.05.

In this work, MDA concentration was used as a marker of oxidative stress. MDA contents were measured in shoot and root tissues of *in vitro* cultured intact plants and in micropropagated shoots, cultured without root system, after 10, 20, and 30 days of treatment (Figure 3). In shoot tissue, either from intact plants or micropropagated shoots, MDA levels significantly increased with the imposition of osmotic stress (ranging from 2,0- to 3,0-fold increase 10 days after the onset of experiments, in shoots of entire plant under moderate stress and microshoots under severe stress, respectively, in comparison to controls). At day 30, increased levels of MDA were found only in shoots from intact plants cultured under severe stress conditions.

On the contrary, in the case of root tissue (Figure 3(b)), higher levels of MDA were measured in control plants 10 and 30 days after the onset of the experiment. At day 20, a significant increase in MDA levels was observed in roots of plants subjected to moderate stress (1,7-fold increase with respect control plants). 

### 3.3. Antioxidant Enzyme Activity

**Figure 4:**
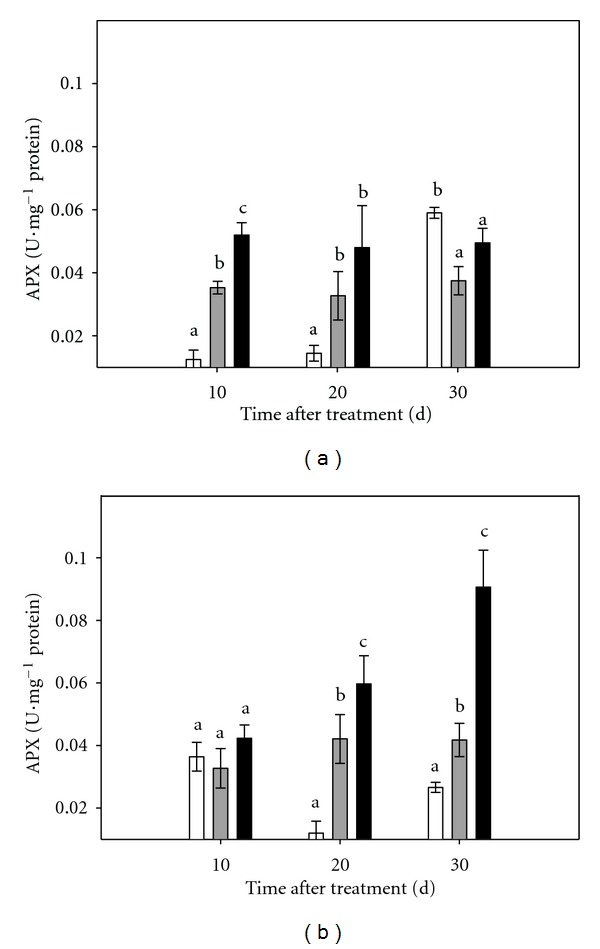
Effect of osmotic stress on APX activity in Carrizo citrange plants cultured *in vitro*. (a): aerial part of intact plants; (b): root tissue of intact plants. Control plants (white bar), plants cultured under moderate (grey bar) or severe (black bar) osmotic stress conditions. Each point corresponds to the average ± standard error of six independent determinations. Different letters denote statistical significance at *P* < 0.05.

**Figure 5:**
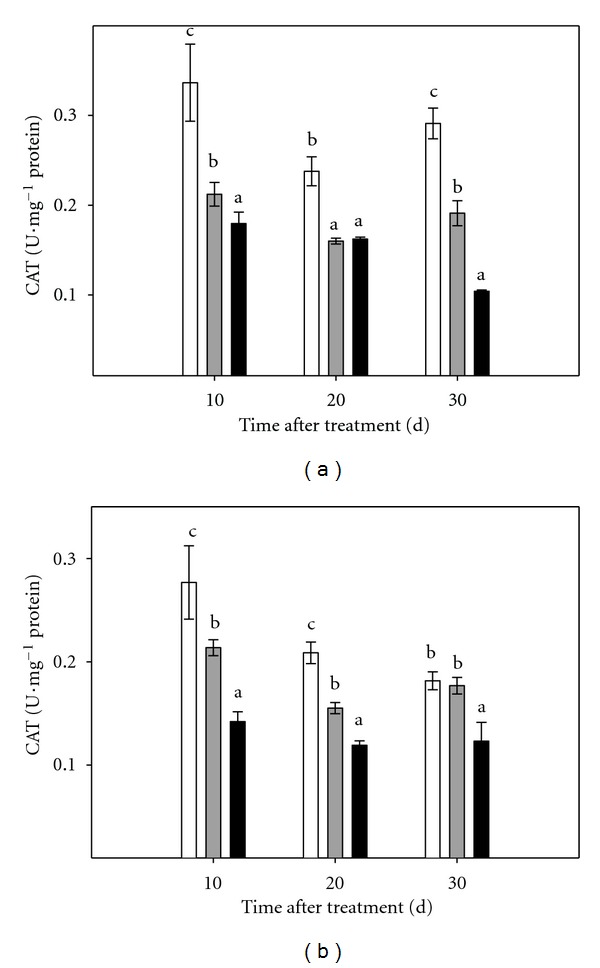
Effect of osmotic stress on CAT activity in Carrizo citrange plants cultured *in vitro*. (a): aerial part of intact plants; (b): root tissue of intact plants. Control plants (white bar), plants cultured under moderate (grey bar) or severe (black bar) osmotic stress conditions. Each point corresponds to the average ± standard error of six independent determinations. Different letters denote statistical significance at *P* < 0.05.

**Figure 6:**
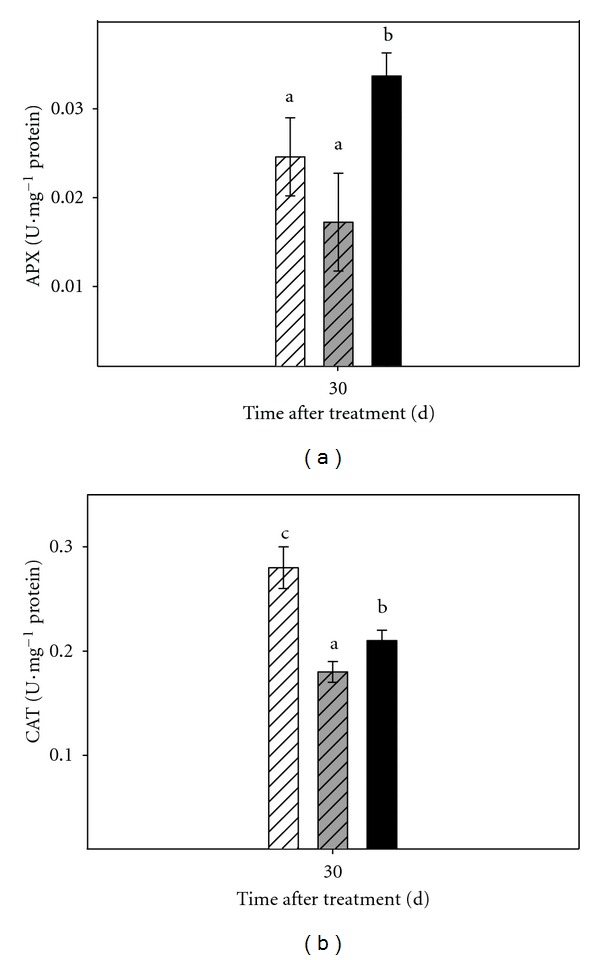
Effect of osmotic stress on enzymatic activity in micropropagated Carrizo citrange shoots. (a): APX activity; (b): CAT activity. Control plants (white bar), plants cultured under moderate (grey bar) or severe (black bar) osmotic stress conditions Each point corresponds to the average ± standard error of six independent determinations. Different letters denote statistical significance at *P * < 0.05.

Antioxidant enzyme activities in intact plants and micropropagated shoots are shown in Figures 4, 5, and 6.

APX activity increased, both in the aerial part and in the roots of plants subjected to moderate or severe stress. The differences were statistically significant between treatments from the first sampling in the case of the aerial part (2.0- and 2.2- fold increase with respect control plants in moderate and severe stress, resp). This pattern was observed throughout all the experimental period (Figure 4(a)). In the root tissue, there was no difference between stressed and control plants at the beginning of the experiment. However, ten days later, APX activity was significantly higher in roots of plants cultured under stress conditions. At day 30, APX activity drastically increased in roots of plants cultured on PEG150, reaching values 3.5 times higher than controls. Roots of plants cultured in PEG075 exhibited a moderate increase in APX activity but still significantly higher than that observed in roots of control plants (Figure 4(b)).

When subjected to osmotic stress (both conditions), lower levels of CAT activity were measured in all assayed plant material, being significantly lower than values recorded in controls, except in the case of roots of plants cultured 30 days under moderate stress conditions, that exhibited values similar to controls (Figure 5). 

Enzymatic activity was determined in micropropagated shoots 30 days after the onset of the experiment (Figure 6), APX activity was higher in plants subjected to severe stress treatment. No differences were detected between shoots cultured in moderate stress and control conditions. On the contrary, in the case of CAT, control shoots exhibited CAT activities higher than those determined in stressed ones. 

### 3.4. Proline Content

**Figure 7:**
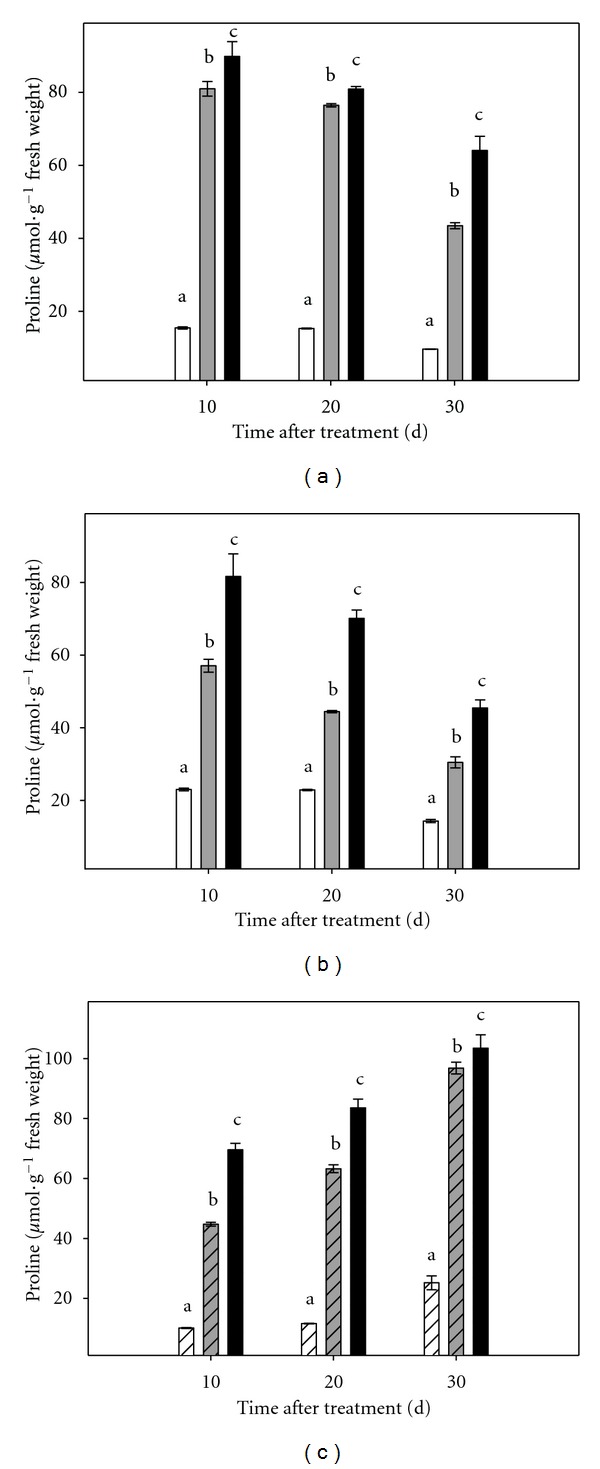
Effect of osmotic stress on proline content in Carrizo citrange plants cultured *in vitro*. (a): aerial part of intact plants; (b): root tissue of intact plants; (c): micropropagated shoots. Control plants (white bar), plants cultured under moderate (grey bar) or severe (black bar) osmotic stress conditions. Each point corresponds to the average ± standard error of six independent determinations. Different letters denote statistical significance at *P* < 0.05.

As shown in Figure 7, as a result of the imposed stress, there was a significant accumulation of proline from the first day of measurement, both in intact plants (in shoot and root tissues) and in micropropagated shoots. Proline content in the aerial part of stressed plants increased to very high levels (that varied from 4.5-fold to 6.6-fold increase). The pattern of proline accumulation in root tissue was similar to that described for shoots, with high levels in stressed plants ranging from 1.9- to 3.5-fold increase with respect to controls. 

Proline contents in micropropagated shoots cultured under stress conditions were significantly higher than those determined in controls throughout the experimental period.

In all assayed plant material, proline levels were proportional to the stress severity (Figure 7).

### 3.5. Hormone Content

**Figure 8:**
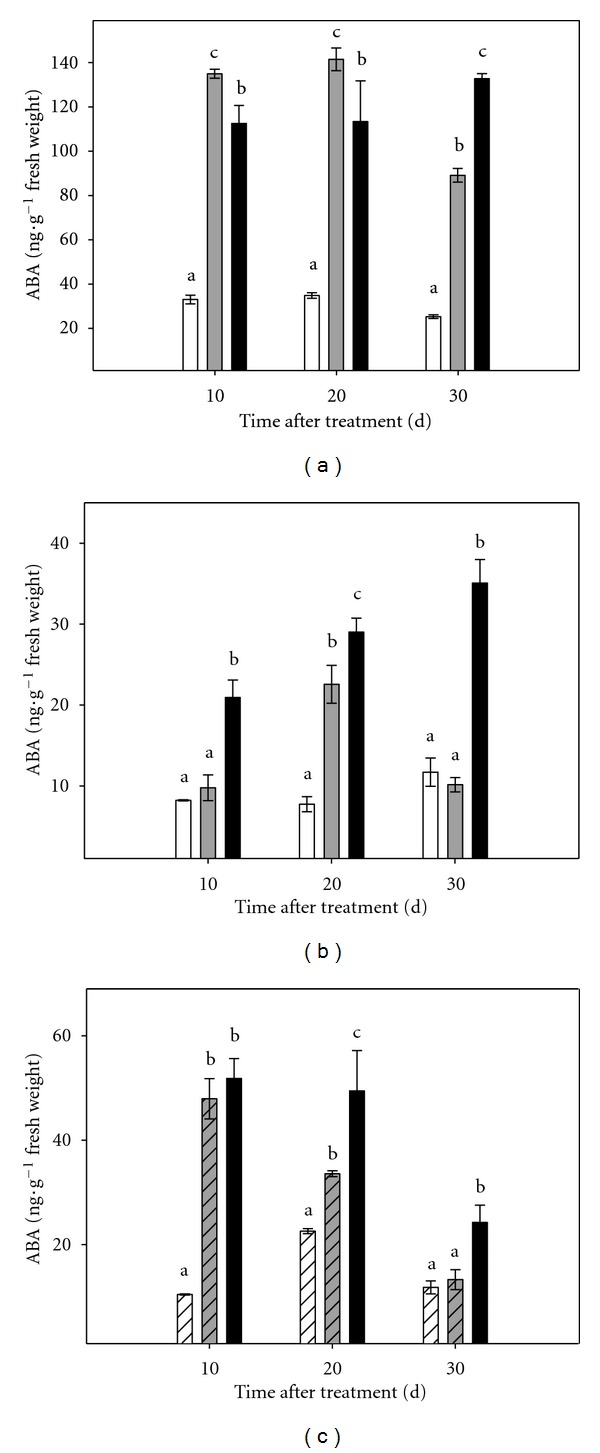
Effect of osmotic stress on ABA content in Carrizo citrange plants cultured *in vitro*. (a): aerial part of intact plants; (b): root tissue of intact plants; (c): micropropagated shoots. Control plants (white bar), plants cultured under moderate (grey bar) or severe (black bar) osmotic stress conditions. Each point corresponds to the average ± standard error of six independent determinations. Different letters denote statistical significance at *P* < 0.05.

Throughout the experimental period, ABA levels were higher in shoots than in roots (Figures 8(a) and 8(b)). Plants under stress showed foliar ABA concentrations higher than those observed in control plants. After 10 and 20 days of stress treatment, leaves of plants subjected to moderate stress had higher concentrations of ABA, (4.0-fold increase with respect to controls, in both cases), than those subjected to severe stress (3.3- and 3.2-fold increase). This situation was reversed at day 30, when plants grown in the PEG150 medium showed ABA concentrations higher than those grown in the PEG075 medium (5.2-fold versus 3.5-fold increase with respect to controls). ABA concentration in roots of plants subjected to severe stress treatment was higher than in controls throughout the experimental period (Figure 8(b)). 

As described for entire plants, ABA content was higher in micropropagated shoots subjected to osmotic stress than in controls (Figure 8(c)). The highest values were observed, throughout the experimental period in microshoots cultured under severe stress conditions.

**Figure 9:**
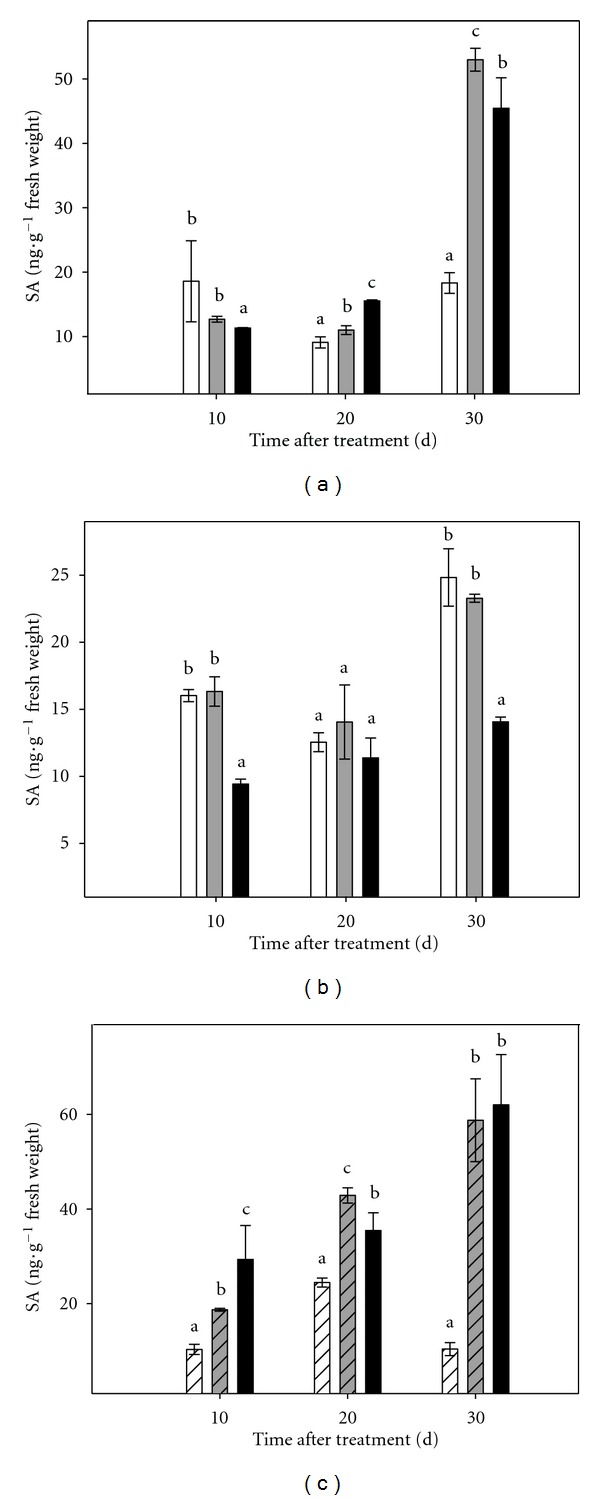
Effect of osmotic stress on SA content in Carrizo citrange plants cultured *in vitro*. (a): aerial part of intact plants; (b): root tissue of intact plants; (c): micropropagated shoots. Control plants (white bar), plants cultured under moderate (grey bar) or severe (black bar) osmotic stress conditions. Each point corresponds to the average ± standard error of six independent determinations. Different letters denote statistical significance at *P* < 0.05.

The content of SA was significantly higher in the aerial part of plants cultured under stress conditions from day 20 of experiment (Figure 9(a)). At day 30, differences increased drastically, reaching values 2.5 and 2.9 times higher than controls in plants under severe and moderate stress respectively. In root tissue, plants cultured under severe stress conditions exhibited, throughout the experimental period, lower concentrations of SA than those cultured under moderate stress or in control conditions, except at day 20 (Figure 9(b)). No differences were found in SA levels in plants cultured in control and PEG75 media.

The osmotic stress caused an increase in endogenous SA levels in micropropagated shoots throughout the experimental period (Figure 9(c)). At the end of the experimental period, there was a marked increase in the levels of this hormone in shoots cultured under stress (5.6- and 5.9-fold increase in shoots subjected to moderate and severe stress, resp.).

**Figure 10:**
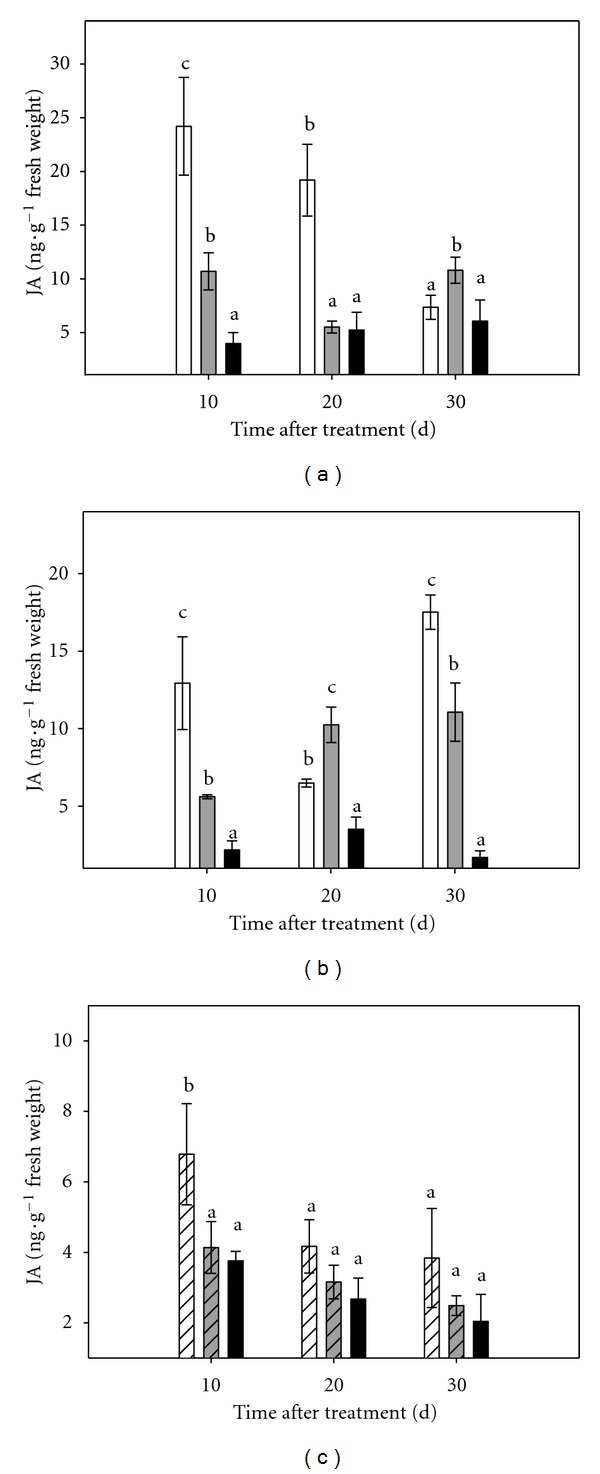
Effect of osmotic stress on JA content in Carrizo citrange plants cultured *in vitro*. (a): aerial part of intact plants; (b): root tissue of intact plants; (c): micropropagated shoots. Control plants (white bar), plants cultured under moderate (grey bar) or severe (black bar) osmotic stress conditions. Each point corresponds to the average ± standard error of six independent determinations. Different letters denote statistical significance at *P * < 0.05.

Ten days after the onset of the experiment, control plants showed JA contents, both in leaf and root tissues, significantly higher than those measured in plants cultured under moderate or severe stress conditions (Figure 10). From this point, JA concentrations erratically varied throughout the experimental period both in roots and shoots. 

The endogenous JA levels in stressed micropropagated shoots were also significantly lower than in controls at day 10. After this, the values recorded for this hormone were similar between control and stressed plants (Figure 10(c)).

## 4. Discussion

Nowadays, *in vitro* culture of plant tissues, allow us to reproduce in laboratory conditions new possibilities for the study of processes that cannot be addressed in plants grown in greenhouses or field conditions [30]. In the field, plants are exposed to variable biological and environmental conditions that can make some basic studies very difficult. *In vitro* plant tissue culture has proved to be a valuable tool for the study of the biochemical processes involved in the response of citrus to a singular abiotic stress condition without the interference of any other factor [9].

In a similar manner to what was observed in previous experiments conducted *ex vitro* [6], *ex vitro *culturing of plants or shoots under osmotic stress conditions caused yellowing and browning of leaves, being the rate of deleterious symptom onset proportional to the severity of the imposed stress. The system also allowed the study of oxidative damage induced by osmotic stress and it was noted that its incidence was higher in the aerial part of plants, which is consistent with previous studies made in plants grown in greenhouses under conditions of continuous flooding of the substrate [17]. In both systems, *in vitro* and *ex vitro*, aerial tissues accumulated higher amounts of MDA, an indirect marker of oxidative damage. When shoots were cultured without the root system, a similar pattern of accumulation was observed, a high increase in MDA content was detected in stressed tissues during the first 20 d of treatment. These values contrast with previous data obtained in a similar system where CC microshoots were cultured under salt stress conditions. In that case, MDA content was similar in stressed and control plants throughout the entire experimental period [9]. Under saline stress conditions, *in vitro* cultured CC shoots accumulated high levels of Cl^−^ ions and exhibited the characteristic necrosis in leaf tissue. This, together with the absence of MDA accumulation, allowed us to conclude that there was no correlation between foliar damage and oxidative stress in the case of that specific abiotic stress. 

It has been proposed that the lack of antioxidant response in *ex vitro* growing plants of the salt tolerant citrus rootstock Cleopatra mandarin (CM) is due to a restricted chloride uptake due to its particular anatomical and physiological characteristics [31]. In our previous work [9] it was proved that under salt stress conditions, *in vitro* microshoots of both genotypes, salt tolerant and sensitive (CM and CC respectively), exhibited the same pattern of Cl- intoxication and, as a consequence, the same foliar damage. In both genotypes oxidative damage in leaf tissue was no detected, as no MDA accumulation was recorded throughout the stress treatment. On the contrary, as described in the present work, when CC microshoots were subjected to osmotic stress, the occurrence of leaf tissue damage was concomitant with the increase in oxidative damage. These results reinforce the idea that different abiotic stresses cause different physiological responses in citrus plants.

Proline concentration increased in all studied plant material when plants or micropropagated shoots were cultured in media supplemented with PEG-6000. It has been reported that, in *ex vitro* conditions, proline levels increased in citrus leaves in response to salt stress conditions [2, 5], soil flooding [17] and drought [20]. High proline levels have been correlated with tolerance to different abiotic stresses, such as drought, salinity and high temperatures [20, 32]. In transgenic “Swingle” citrumelo plants over-expressing P5CSF129A gene, which codes for the key-enzyme for proline synthesis, it seems that the high proline content mitigates the effect of ROS, by directly scavenging free radicals and by activating antioxidant systems [18]. However, the putative protective effect of proline is not very effective in the conditions described in this work because plants subjected to increasing concentrations of PEG-6000 accumulated significant amounts of proline in parallel with the increase of oxidative damage. These observations are consistent with those shown *ex vitro* in citrus grown under conditions of continuous flooding of the substrate [17], where the highest concentrations of this amino acid were found in the most damaged genotypes. Therefore, despite the controversy of the effectiveness of the accumulation of endogenous proline in wild-type plants under stress as a protective mechanism, it seems that the increase of this metabolite in citrus leaves could be considered as a good stress marker.

Data presented here also demonstrate that root tissue is not necessary for osmotic stress to cause a high increase in proline content as high levels of this amino acid were detected in micropropagated shoots cultured in media supplemented with PEG. Different response was observed under salt stress conditions and in that experimental system no proline accumulation was detected in stressed microshoots cultured without roots [9]. This adds new evidence confirming that plant is differently affected by the osmotic and toxic components of salt stress.

It is known that the activities of several enzymes are affected by stress conditions; as a general pattern SOD, CAT and APX activities increase in response to different abiotic stresses, such as water deficit [33] or flooding [17]. Under osmotic stress conditions, plants grown *in vitro* are able to activate, their antioxidant machinery. As it could be expected, APX activity increased, not only in roots and leaves of intact plants but also in micropropagated shoots subjected to stress conditions. On the contrary, CAT activity decreased both, in plants and in micropropagated shoots grown in medium supplemented with PEG-6000. Similar trend was observed in CC plants cultured *ex vitro* under stress conditions in response to flooding of the substrate [34] and in citrumelo plants, when transgenic plants over-expressing the *P5CSF129A* gene were cultured under osmotic stress conditions [18]. The increase in APX and the reduction in CAT activities as a consequence of the osmotic stress treatment in *in vitro* cultured CC plants, concomitant with the high increase detected in proline contents may suggest that CAT is not participating in the protection against oxidative stress. Data also show that in *in vitro* conditions, plants retain their antioxidant machinery intact even when micropropagated shoots are cultured without root system. 

Osmotic stress caused a significant accumulation of ABA in both, intact plants and micropropagated shoots. This agrees with the data obtained plants grown in greenhouse and field [5, 29, 35] under conditions of water stress, high salinity and flooding of the substrate and reinforces the role of ABA as a mediator of plant responses. It is important to point out that citrus leaves accumulate the same amount of ABA under osmotic stress conditions even when cultured without root system. On the contrary, no accumulation of ABA was observed when CC micropropagated shoots were cultured *in vitro*, under salt stress conditions [9]. On the view of these results it can be suggested that the pattern of signaling is dependent on the type of adverse condition that plant has to cope with. 

The hormone SA is considered to be involved in plant resistance to several plant pathogens [36] and also appears to have an important role in plant response to oxidative stress [37]. The increased SA content in the aerial part of the plant at the end of the experimental period support this hypothesis, as it is concomitant with the occurrence of severe oxidative damage in this tissue. 

The decrease in JA levels, observed throughout the experimental period in both leaf and root tissue of intact plants and in micropropagated shoots, is compatible with previous data which showed that JA acts as mediator between the perception of the stress and the induction of physiological responses and, therefore, its action should be early [29, de Ollas et al., 2012, unpublished data]. Although it was not the main point of this work, it is possible that the first data collection point was carried out too late to find a possible transient increase in JA concentration. However, the data presented here show a decreased levels of JA in stressed plants, similar to that described previously in citrus plants cultured *ex vitro* under abiotic stress conditions [29]. 

It has been reported that, in plants grown in field, the responses to water and salt stress are essentially identical [38]. When cultured *in vitro* without roots, CC shoots growing under osmotic stress conditions shown a significant increase of MDA, proline, and ABA levels. On the contrary, no increase in the endogenous concentrations of these compounds was observed when cultured under saline stress conditions [9]. These results evidence that the mechanisms of response of CC shoots to saline or osmotic stress are different. There is an organ-dependent response to stress: in salt stress conditions it is necessary the presence of the roots for the signaling of stress to the aerial part. On the contrary, the presence of this organ is not necessary to modulate the response of shoots to osmotic stress caused by PEG6000. Therefore, the extrapolation of the information obtained studying the effects of an abiotic stress factor to that obtained when considering other stress factors should be avoided. On the other hand, it is important to note that the organ involved in the stress perception/signaling depends on the type of adverse condition to which plants/shoots are subjected.
